# Maternal imprinting on cognition markers of wild type and transgenic Alzheimer’s disease model mice

**DOI:** 10.1038/s41598-018-24710-7

**Published:** 2018-04-24

**Authors:** Marta Zamarbide, Francisco J. Gil-Bea, Paul Bannenberg, Eva Martínez-Pinilla, Juan Sandoval, Rafael Franco, Alberto Pérez-Mediavilla

**Affiliations:** 1 Neuroscience Department, Center for Applied Medical Research (CIMA), University of Navarra, Pamplona, Spain; 2 Laboratory of Molecular Neurobiology, Department of Biochemistry and Molecular Biomedicine, Faculty of Biology, University of Barcelona, Barcelona, Spain; 3 Centro de Investigación en Red, Enfermedades Neurodegenerativas (CIBERNED), Instituto de Salud Carlos III, Madrid, Spain; 4 Department of Biochemistry and Genetics, School of Sciences, University of Navarra, Pamplona, Spain; 5 Laboratory of personalized medicine, Epigenomics Unit, Medical Research Institute La Fe, Valencia, Spain

## Abstract

The risk of suffering from Alzheimer’s disease (AD) is higher in individuals from AD-affected mothers. The purpose of this investigation was to study whether maternal transmission might produce AD-related alterations in progenies of mice that do not have any genotypic alteration. We used cognitively-intact mothers harbouring in heterozygosity the transgene for overexpressing the Swedish double mutant version of the human amyloid precursor protein (hAβPPswe). The phenotype of the offspring with or without the transgene resulting from crossing young Tg2576 females with wild-type males were compared with those of the offspring resulting from crossing wild-type females with Tg2576 males. The hAβPPswe-bearing offspring from Tg2576 mothers showed an aggravated AD-like phenotype. Remarkably, cognitive, immunohistochemical and some biochemical features displayed by Tg2576 heterozygous mice were also found in wild-type animals generated from Tg2576 females. This suggests the existence of a maternal imprinting in the wild-type offspring that confers a greater facility to launch an AD-like neurodegenerative cascade. Such progeny, lacking any mutant amyloid precursor protein, constitutes a novel model to study maternal transmission of AD and, even more important, to discover early risk markers that predispose to the development of AD.

## Introduction

Two different types of Alzheimer’s disease (AD), which is the most common cause of dementia, are identified on the basis of pathologic, genetic and molecular evidence. The early-onset Alzheimer’s disease (EOAD) is due to mutations of human amyloid-β precursor protein (hAβPP) or presenilin genes. Late-onset Alzheimer’s disease (LOAD), the most common form of the disease, is not linked to specific gene alterations and its aetiology is caused by a complex interaction between genetic and life-style factors. After age, the second most important risk factor for LOAD is having a parent with the disease^1,2^. In particular, it is assumed that maternal inheritance contributes more than parental inheritance to the risk of suffering AD. Therefore, different studies with LOAD cases have consistently found that maternal AD history is 2–3 times more abundant than the paternal one^3–6^. Honea *et al*. described a correlation between maternal family history of AD (MH) and lower grey matter volume in AD vulnerable brain regions^7^. Maternal family history in LOAD has been also related with a reduced activity of the key mitochondrial enzyme, cytochrome oxidase^8^. Moreover, mutations in the X chromosome and mitochondrial DNA are also behind the consequences of maternal inheritance^9^. Less studied is whether maternal transmission of non-(nuclear)genetic components constitutes a risk factor in vertical AD transmission.

High blood pressure, diabetes and hypercholesterolemia are among the variety of risk factors that have been linked to LOAD. A common factor in the disease is oxidative stress, whose reduction by antioxidants is considered promising to combat AD. Oxidative stress markers in the cerebrospinal fluid of AD patients^10^ reflect pro-oxidant conditions and alterations in cerebral structures. In fact, the overall data point to oxidative damage as an early event in AD and to a relevant role of mitochondria in AD pathogenesis^11^. Oxidative modified nucleic acids have been found in AD brains^12^ and increased lipid peroxidation is already observable in the early stages of the disease^13^. Acrolein, is a product of lipid peroxidation that causes oxidative stress in brain mitochondria^14,15^, is upregulated in AD brains^16^ and is present in more than half of neurofibrillary tangles^17^. The best studied EOAD -associated mutation is the Swedish double mutation in the hAβPP gene (hAβPPswe)^18^. This mutation has been instrumental to develop the Tg2576 transgenic mouse model, which overexpresses hAβPPswe. Cognitive alterations in the Tg2576 mouse appear from nine months of age and, interestingly, it correlates with marked alterations in the level of mitochondrial proteins^19^.

The aim of this work was to assess the risk of displaying cognitive deficits associated to be offspring of Tg2576 females. From behavioural and biochemical assays, we demonstrate that wild type progeny from Tg2576 females have some of the most characteristic features of AD. The generated animal models, WT and heterozygous Tg2576 animals with maternal imprinting (WT_M_ and Tg2576_M_), show promise to understand AD maternal transmission and to discover early risk markers that predispose to develop AD.

## Results

### Behavioral impairment in WT_M_ mice

**Figure 1:**
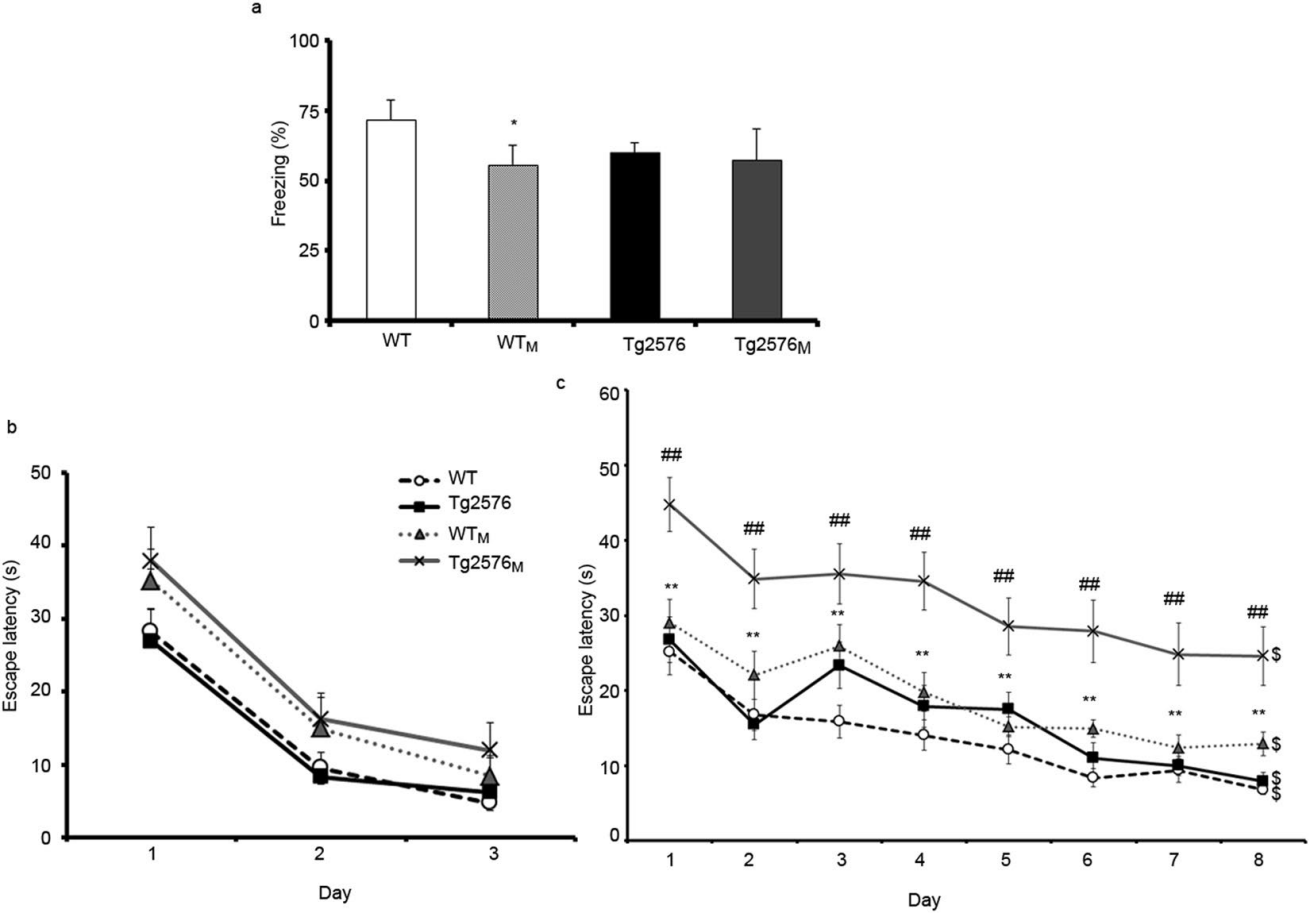
Fear conditioning and MWM tests in the four animal groups (WT, WT_M_, Tg2576 and Tg2576_M_). The results are expressed as the mean ± SEM (n = 10–12). Statistical significance was analysed by two-way ANOVA with Tukey’s *post-hoc* test. Panel a, percentage of freezing in the fear conditioning test performed in 4-month-old animals. Panels b-c, escape latency of 8-month-old mice in the visible (**b**) and hidden-platform (**c**) of the MWM. *p < 0.05, **p < 0.01 respect to WT; ^##^p < 0.01 respect to Tg2576; ^$^p < 0.05 day 1 respect to day 8.

The cognitive status of the different animal groups, consisting of WT and Tg2576 offspring from wild type (non-hAβPPswe) or hAβPPswe-overexpressing mothers, was assessed at four months using a conditioned fear protocol. The choice of this age is based on previous data showing impairment in this test in four-month-old Tg2576 mice^20^. WT_M_ animals exhibited impaired fear conditioned memory as they showed lower freezing times than WT mice (F_3,56_ = 7.6; p < 0.05 Fig. 1a).

Likewise, we evaluated the spatial memory of the animals by the Morris Water Maze test (MWM). The test was performed at an age of 8 months, when it has been already demonstrated that the Tg2576 mice (coming from a WT female and a Tg2576 male) do not yet display deficits in spatial memory. Figure 1b indicates that all animals could learn in the training sessions. Escape latencies were progressively reduced in all groups, with differences between day 1 and day 3. Escape latencies to hidden platform of both Tg2576_M_ and WT_M_ mice were significantly longer than those of their corresponding controls (WT and Tg2576, F_3,348_ = 10.73; p < 0,01) (Fig. 1c). It is noteworthy that the performance of WT_M_ mice was significantly worse than that of WT animals. Therefore, maternal transmission resulted in affected cognitive function in the WT_M_ group and accelerated the cognitive decline in the Tg2576_M_ mice.

### WT_M_ animals display some AD histopathological features

**Figure 2:**
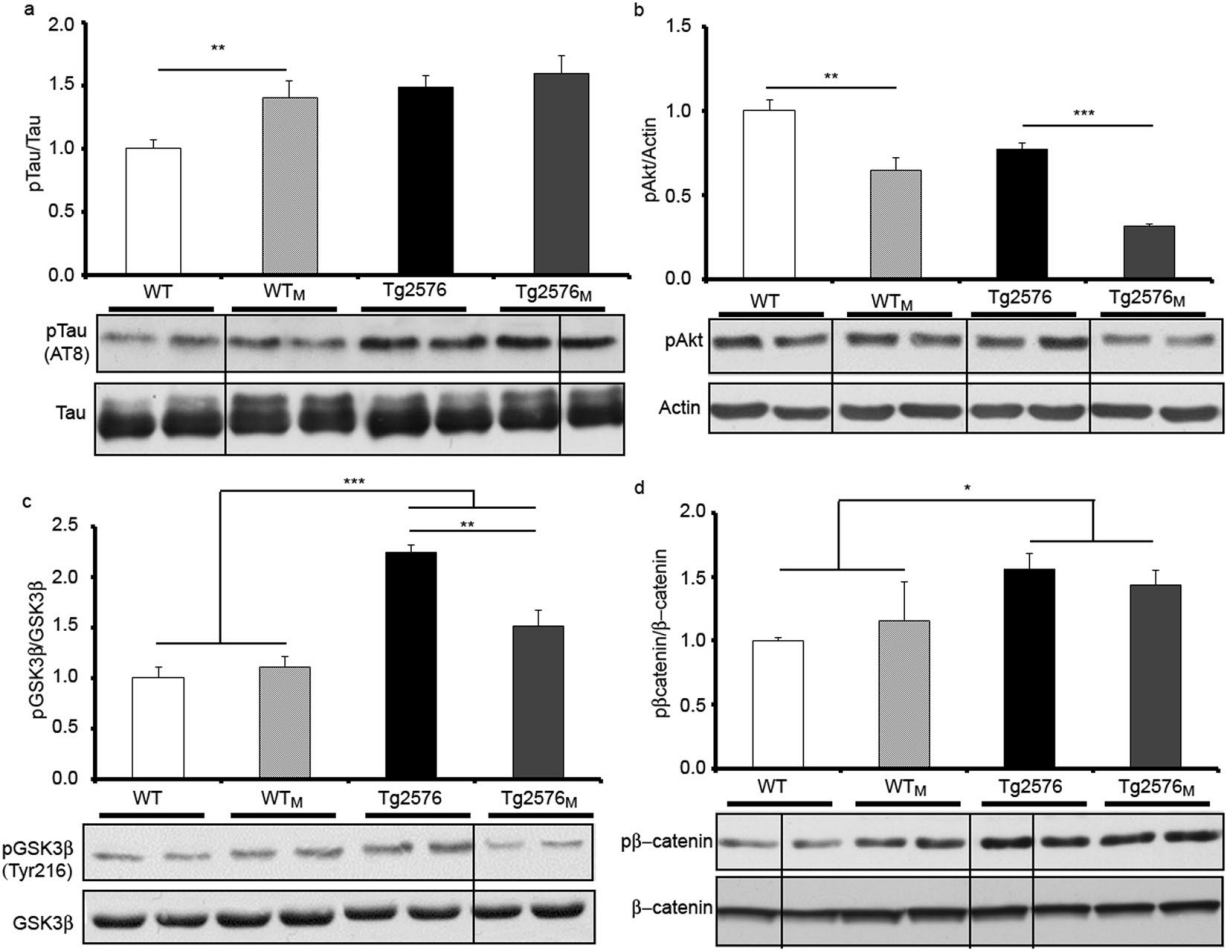
Levels of Tau, pTau and of proteins involved in regulating Tau phosphorylation. Results from Western blot of hippocampal membrane extracts from WT, WT_M_, Tg2576 and Tg2576_M_ mice are displayed. We show representative lanes from representative blots (full-length blots are presented in Supplementary Figure 1) and the quantification of immunolabeled bands. The results (fold over control –WT-) are expressed as the mean ± SEM (n = 4). Statistical significance was analysed by two-way ANOVA with Tukey’s *post-hoc* test (**a**) pTau *versus* Tau. (**b**) pAkt *versus* actin. (**c**) pGSK3β (Tyr216) *versus* GSK3β. (**d**) pβcatenin *versus* β-catenin. *p < 0.05, **p < 0.005, ***p < 0.0001.

**Figure 3:**
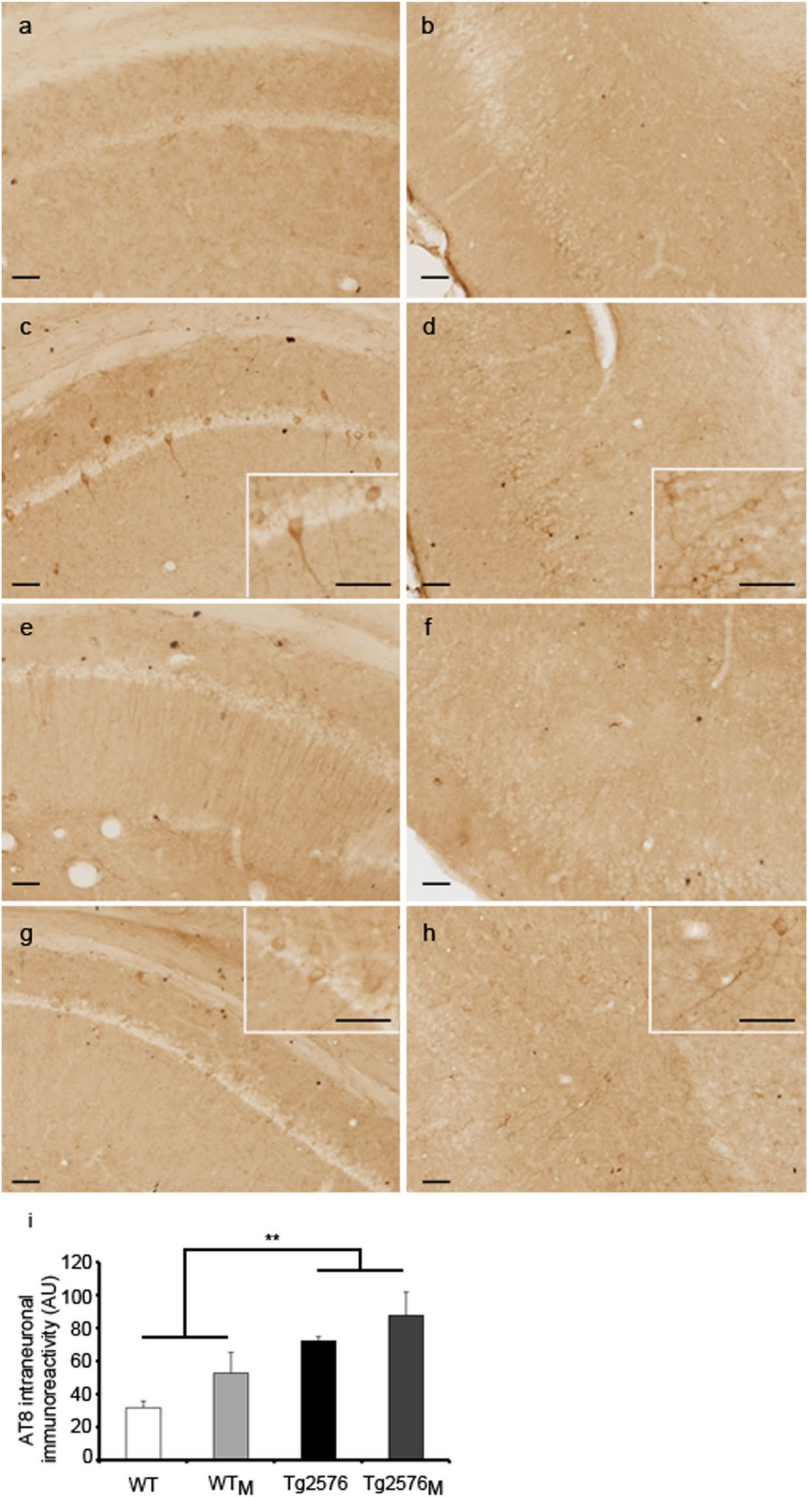
Immunohistochemistry using the AT8 antibody in hippocampal (**a**,**c**,**e**,**g**) and entorhinal cortex (**b**,**d**,**f**,**h**) sections from WT (**a**,**b**), WT_M_ (**c**,**d**), Tg2576 (**e**,**f**) and Tg2576_M_ (**g**,**h**) mice. Scale bars: 50 µm. Quantification of AT8 staining per hippocampal section (i). Data are the mean ± SEM of six different areas covering the entire hippocampus from two sections on the anatomically comparable plane for each animal (n = 3). Statistical significance was analysed by two-way ANOVA with Tukey’s *post-hoc* test. **p < 0.005.

To identify mechanisms underlying the poor task performance in offspring from AD-affected mothers, a variety of biochemical parameters were analysed in cortical and hippocampal samples, including immunohistochemistry studies to detect hAβPP and pTau in hippocampal slices. As the pTau/Tau balance is a key factor in AD pathology, the relative levels of pTau and of proteins involved in Tau phosphorylation were assessed by immunoblotting in hippocampal samples. The pTau/Tau ratio was not only elevated in Tg2576 and Tg2576_M_ but in WT_M_ animals (F_1,12_ = 12.08; p < 0.01) (Fig. 2a). The fact that the two-way ANOVA analysis shows no interaction between transgenic and maternal imprinting strongly suggests that the increase in pTau in the WT_M_ mice was due to the maternal inheritance. The results obtained when Akt phosphorylation (Ser473), was analysed were similar. Akt phosphorylation (Ser473), which is a relevant parameter in the regulation of pTau levels^21,22^, was decreased in Tg2576_M_ *versus* Tg2576 (F_1,12_ = 23.90; p < 0.001) and in WT_M_ *versus* WT (F_1,12_ = 48.72; p < 0.01); the statistical analysis shows that the effect was due to maternal inheritance and not to being transgenic. It should be noted that Akt phosphorylation was significantly lower in the Tg2576_M_ than in any other group (Fig. 2b). The analysis of Tyr^216^ phosphorylation, which activates GSK3β^23^ and leads to aberrant Tau phosphorylation shows significant interaction (F_3,12_ = 16.56; p < 0.001) between the transgenic and maternal inheritance factors. The activation of GSK3β is enhanced in transgenic animals but at higher levels in Tg2576 than in Tg2576_M_ (F_1,12_ = 65.10; p < 0.01) (Fig. 2c). Moreover, phosphorylation levels of βcatenin, which is targeted by GSK3β for phosphorylation and subsequent degradation^24,25^, were significantly enhanced in the two groups of transgenic animals but without any effect due to maternal transmission (F_1,12_ = 16.677; p < 0.05) (Fig. 2d). In line with the immunoblotting results displayed in Fig. 2a, pTau immunohistochemistry showed that both transgenic and WT_M_ animals exhibited pTau-immunoreactive neurons in hippocampus and entorhinal cortex. Images of AT8 immunoreactivity are shown in Fig. 3a–h and quantification in Fig. 3i. The results show differences in AT8 due to transgenicity (F_1,8_ = 15.54; p < 0.01) and a tendency when maternal transmission was considered.

**Figure 4:**
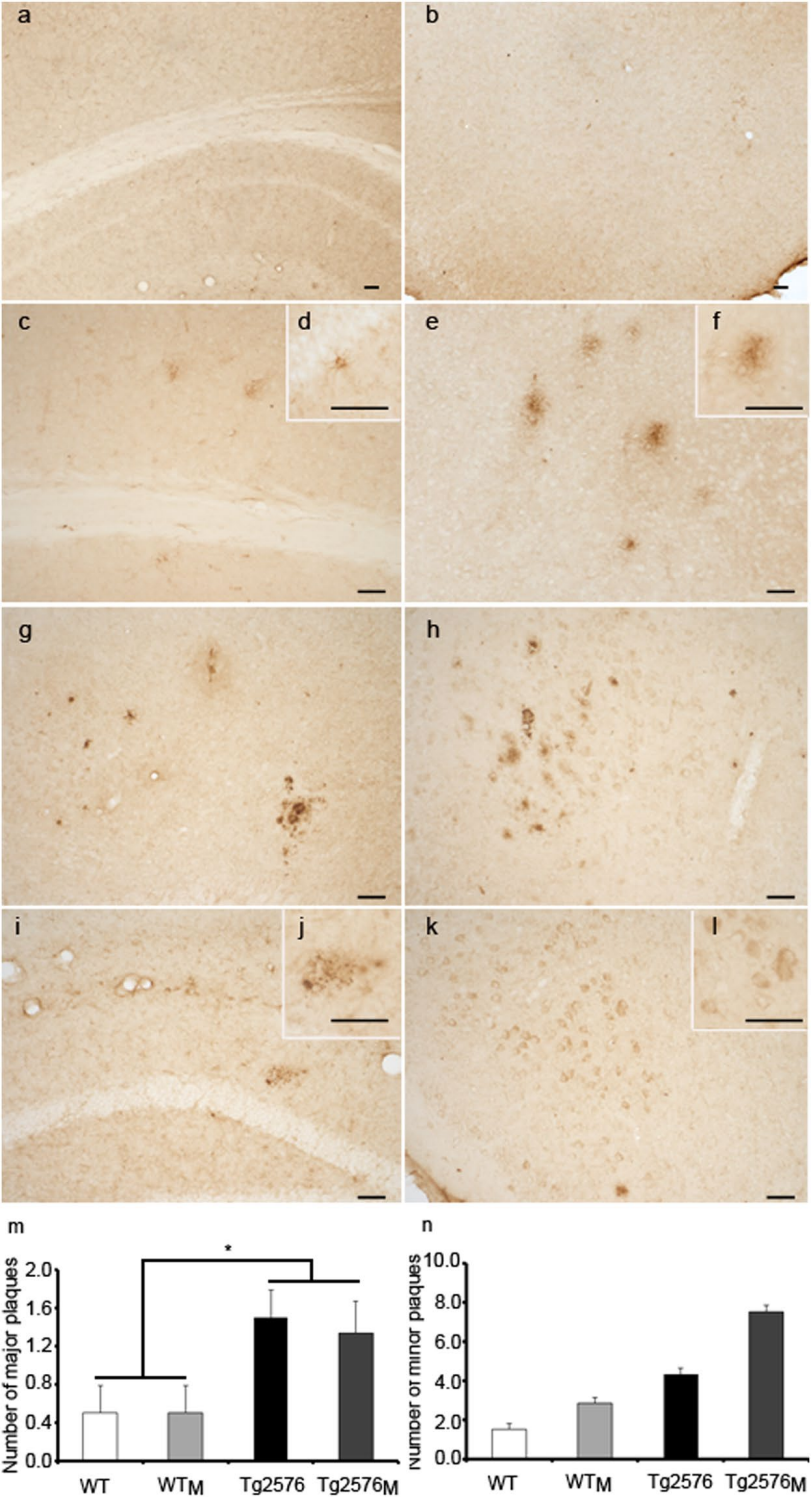
Immunohistochemistry using the 4G8 antibody in sections from WT (**a**,**b**), WT_M_ (**c**–**f**), Tg2576 (**g**,**h**) and Tg2576_M_ (**i**–**l**) mice. Panels a, c, g and i are from hippocampus; panels b, e, h, and k are from entorhinal cortex. Animals used were 8-month-old in panels a-f and i-j and 15-months-old in panels g-h and k-l. Scale bars: 50 μm. Aβ staining was quantified as number of major and minor plaques per hippocampal section (**m-n**). Data are the mean ± SEM. Six different areas covering the entire hippocampus from two sections on the anatomically comparable plane for each animal were analyzed (n = 3). Statistical significance was analysed by two-way ANOVA with Tukey’s *post-hoc* test. *p < 0.05.

**Figure 5:**
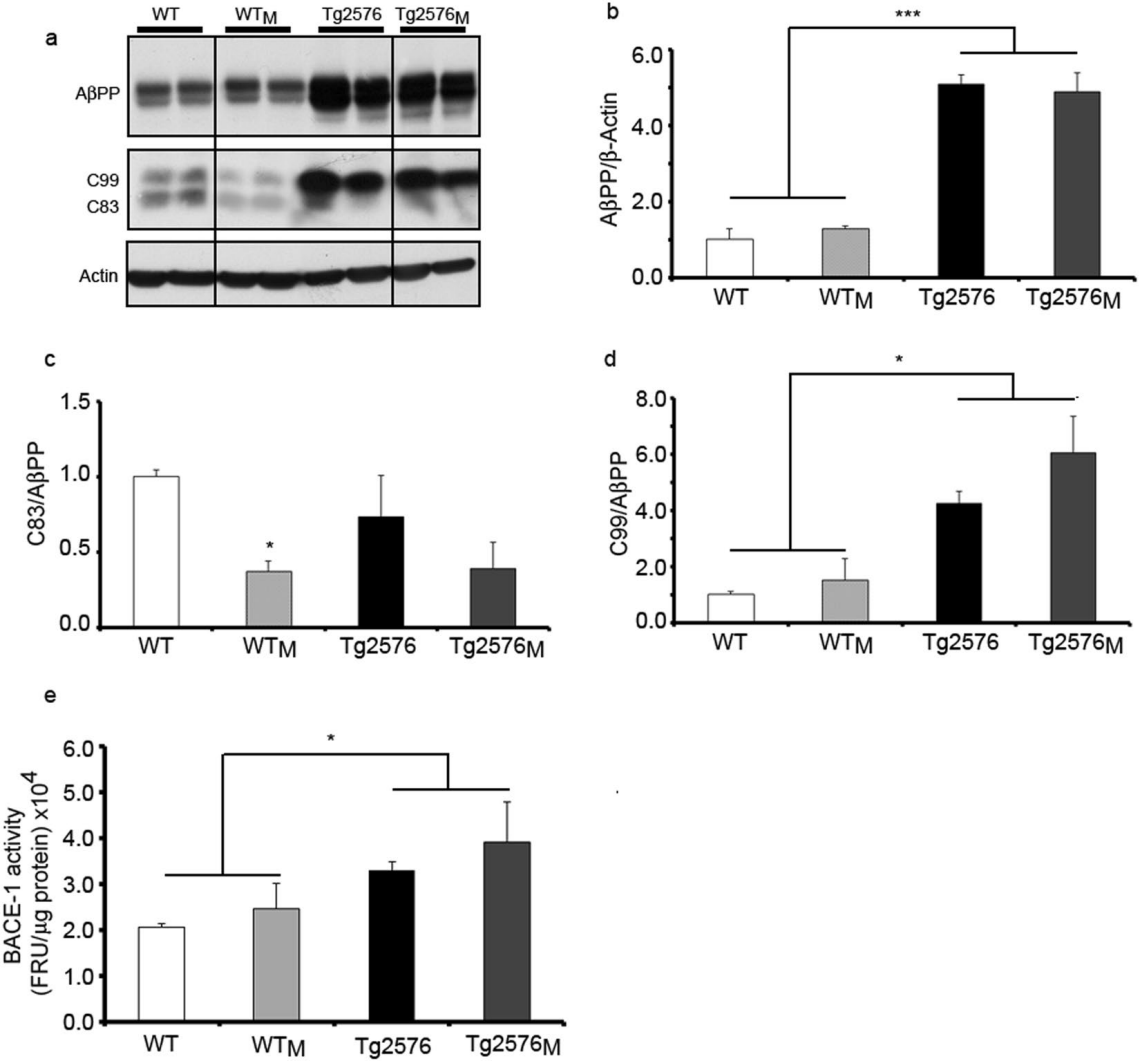
Proteolytic processing of AβPP. (**a**) Representative Western blot of full-length AβPP and AβPP carboxy-terminal fragments, C99 and C83, in total protein cortical extracts. Full-length blots are presented in Supplementary Figure 2. Quantification of immunolabeled bands for AβPP (**b**), C83 (**c**) and C99 (**d**). The results (fold over control –WT-) are expressed as the mean ± SEM (n = 4). (**e**) BACE-1 activity. The activity of BACE-1 was determined as described in Methods. Data are the mean ± SEM (n = 4). Statistical significance was analysed by two-way ANOVA with Tukey’s *post-hoc* test. *p < 0.05, ***p < 0.001.

Finally, the impact of maternal transmission on one of the main hallmarks of the AD pathology, AβPP processing, was assessed by immunohistochemistry (Fig. 4a–l) distinguishing *major* “mature” from *minor* “diffuse” plaques, immunoblotting (Fig. 5a–d) and BACE1 activity (Fig. 5e). The use of the 4G8 antibody, which detects a common human/mouse amyloid-β (Aβ) sequence in the AβPP, confirmed that maternal transmission could give rise to aggregated forms of mouse Aβ both in the hippocampus and entorhinal cortex of WT_M_ and Tg2576_M_. When major plaques were addressed we found an increase due to the transgenic factor and not to the maternal inheritance (F_1,8_ = 10.88; p < 0.05) (Fig. 4m). In the case of minor plaques, maternal inheritance had a clear effect in transgenic animals and, although not significant, a tendency of higher expression in WT_M_ *versus* WT animals was found (Fig. 4n).

These results show that maternal transmission of non-genetic material accelerated plaque deposition in Tg2576_M_ brain and, strikingly, provoked the aggregation of the murine amyloid on WT_M_ mice. Immunoblotting using the CT19 antibody, which detects the C-terminal fragment of both human and murine AβPP, confirmed that the level of AβPP expression was not affected by maternal transmission in either WT or transgenic animals (Fig. 5a,b). Statistical analysis showed that there are no differences in AβPP levels between WT and WT_M_ and neither between Tg2576 and Tg2576_M_. Production of C83 peptide, which results from the non-amyloidogenic processing pathway, was significantly decreased in WT_M_ respect to WT (F_1,12_ = 10.81; p < 0.05); a similar trend without statistical significance was observed in Tg2576_M_ *versus* Tg2576 (Fig. 5c). Production of C99 peptide precursor of Aβ, was significantly higher in the transgenic mice when compared to WT or WT_M_ mice (F_1,12_ = 10.01; p < 0.05), and no significant differences were found between Tg2576 and Tg2576_M_ (Fig. 5d). Due to the tendency of increased C99 expression found by maternal transmission in both WT and transgenic animals, we measured BACE-1 activity. The results (Fig. 5e) were very similar to those obtained for C99 expression, i.e. increase due to transgenicity (F_1,12_ = 11.81; p < 0.05) and no significant changes but a tendency due to maternal transmission.

Considering maternal inheritance, the results showed a similar non-significant tendency as in the C99 expression, while the activity was significantly higher in transgenic versus non-transgenic animals.

### Synaptic plasticity impairment in the offspring of Tg2576 mothers

**Figure 6:**
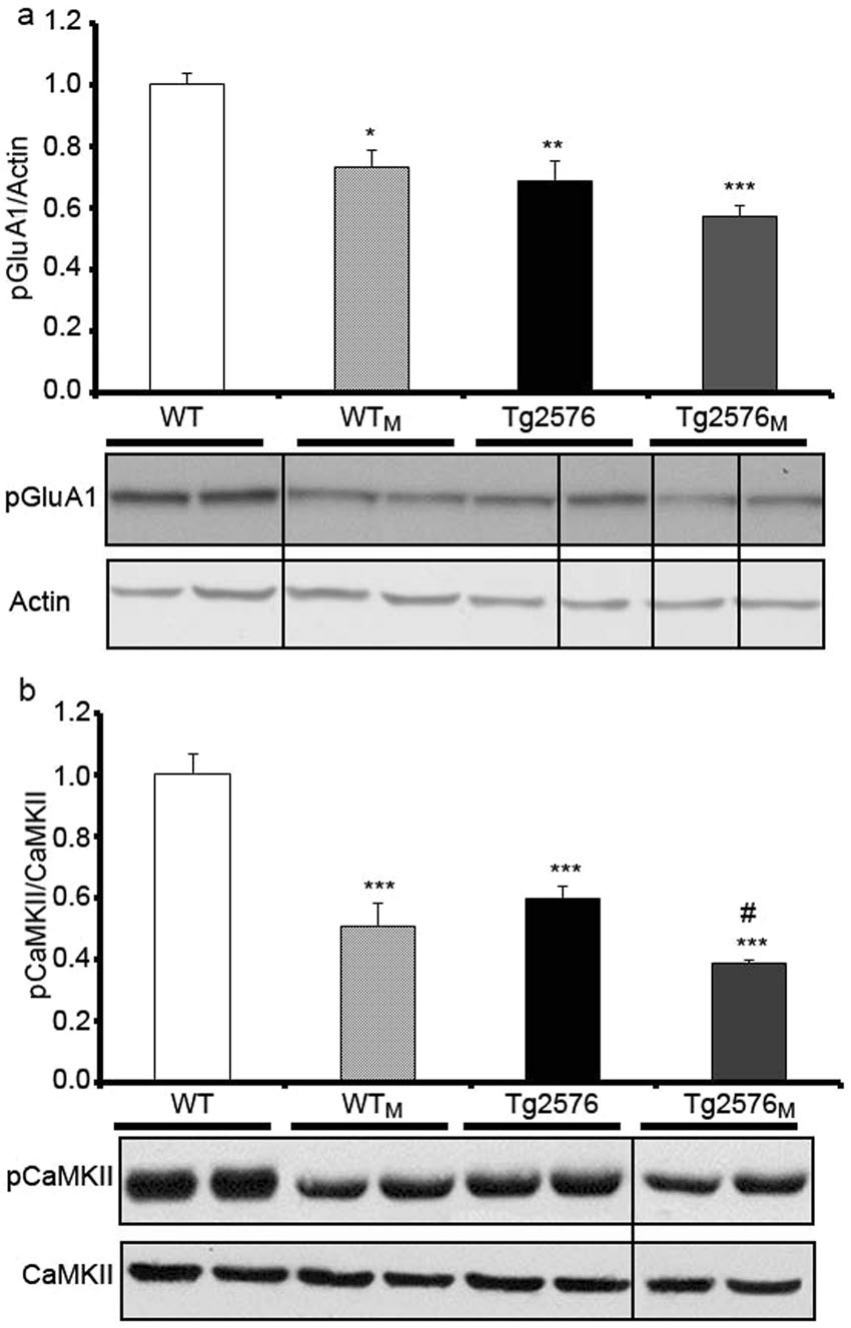
Effect of maternal transmission of non-genetic material on synaptic plasticity markers. The levels of synaptic markers in total protein cortical preparations from WT, WT_M_, Tg2576 and Tg2576_M_ mice were analysed by Western blot. We show representative blots (full-length blots are presented in Supplementary Figure 3) and the quantification of immunolabeled bands. The results (fold over control –WT-) are expressed as the mean ± SEM (n = 4). Statistical significance was analysed by two-way ANOVA with Tukey’s *post-hoc* test. (**a**) pGluA1 AMPA receptor subunit  *versus* actin. (**b**) Phosphorylated CaMK II *versus* total CaMK II. *p < 0.05, **p < 0.005, ***p < 0.0001 respect to WT; ^#^p < 0.05 respect to Tg2576.

Ionotropic AMPA glutamate receptors (GluR) are composed of A1 and A2 subunits whose phosphorylation status is altered in AD^26^. Whereas total levels of A2/3 subunits were not affected (Supplementary Figure 4A), the degree of GluA1 phosphorylation was significantly reduced in all groups when compared with WT animals (F_1,12_ = 10.46; p < 0.05) (Fig. 6a). The phosphorylated and active state of CaMKII, which is key for long-term potentiation and synaptic plasticity^27^, was also reduced in these groups (Fig. 6b). Interestingly, pCaMKII levels were significantly decreased in Tg2576_M_ (compared to Tg2576) (F_1,12_ = 16.21; p < 0.05). The level of PSD95, a marker for post-synaptic densities, was rather similar in the four groups (Supplementary Figure 4b). These findings suggest the possibility of maternal-transmission-related impairment of synaptic function in the cortical tissue.

**Figure 7:**
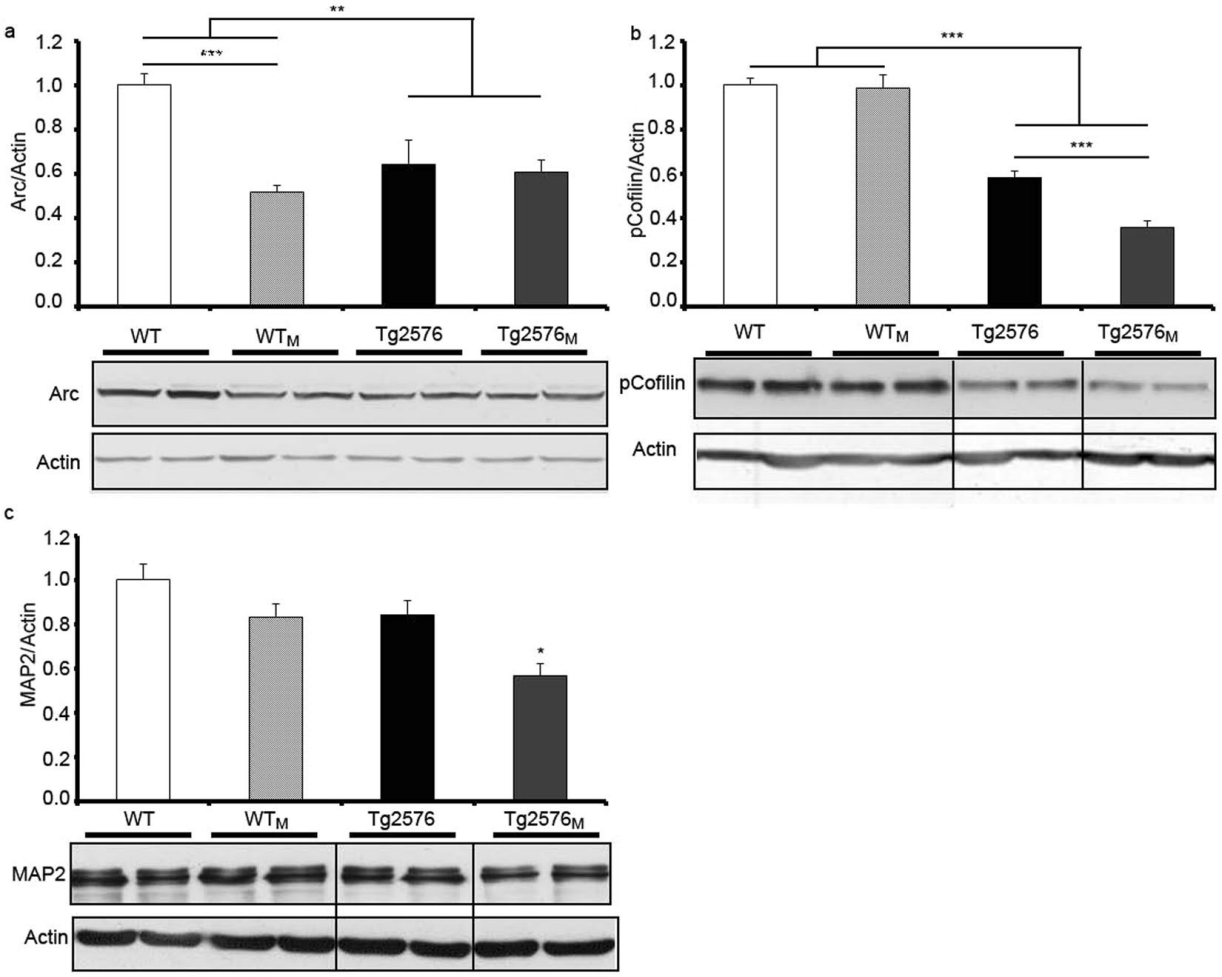
Effect of maternal transmission of non-genetic material on neuronal cytoskeletal markers. The levels of cytoskeleton markers in total protein cortical preparations WT, WT_M_, Tg2576 and Tg2576_M_ mice were analysed by Western blot. Representative blot and quantification of immunolabeled bands for (**a**) Arc *versus* actin, (**b**) pCofilin *versus* actin and (**c**) MAP2 *versus* actin. Full-length blots are presented in Supplementary Figure 5. The results (fold over control –WT-) are expressed as the mean ± SEM (n = 4). Statistical significance was analysed by two-way ANOVA with Tukey’s *post-hoc* test. *p < 0.05, **p < 0.005, ***p < 0.0001 respect to WT; ^###^p < 0.0001 respect to Tg2576.

Microtubules and actin microfilaments are stabilized during neuronal morphogenesis and are critical for intracellular transport and for overall neuronal and spine architecture and dynamics^28,29^. The level of three cytoskeletal protein markers was quantitated in membranes from cortex. Activity-regulated cytoskeleton-associated protein (Arc) is required for memory consolidation through regulation of F-actin expansion thus playing critical roles in synaptic plasticity and memory storage^30^. Arc levels analysed by a two-way ANOVA showed a significant decrease (F_1,12_ = 18.98; p < 0.01) in the transgenic versus WT mice. Also, a *post-hoc* test revealed in WT mice a marked decrease in Arc expression due to the maternal transmission (F_1,12_ = 23.69; p < 0.001) (Fig. 7a). Cofilin is involved in nucleation, polymerization/depolymerization and reorganization of actin. As neurodegenerative stimuli leads to activation of cofilin by dephosphorylation^31^ the p-cofilin/actin ratio was quantified. The results show a decrease in p-cofilin in transgenic animals. The two-way ANOVA analysis also demonstrated a significant effect (F_3,12_ = 23.30; p < 0.001) in the transgenic mice, regardless of which progenitor carried the transgene (Fig. 7b); the post-hoc test revealed significantly lower levels of p-cofilin in Tg2576_M_ mice when compared to Tg2576 animals (F_1,12_ = 605.1; p < 0.001). Together with Tau, the microtubule-associated protein 2 (MAP2) is one of the main microtubule-associated protein in mammalian nerve cells. Interestingly, this protein only decreased significantly in Tg2576_M_ although a similar but no statistically significant tendency was found in WT_M_ and Tg2576 animals (Fig. 7c).

### Mitochondrial VDAC-1 and HXKI levels were affected by maternal transmission

**Figure 8:**
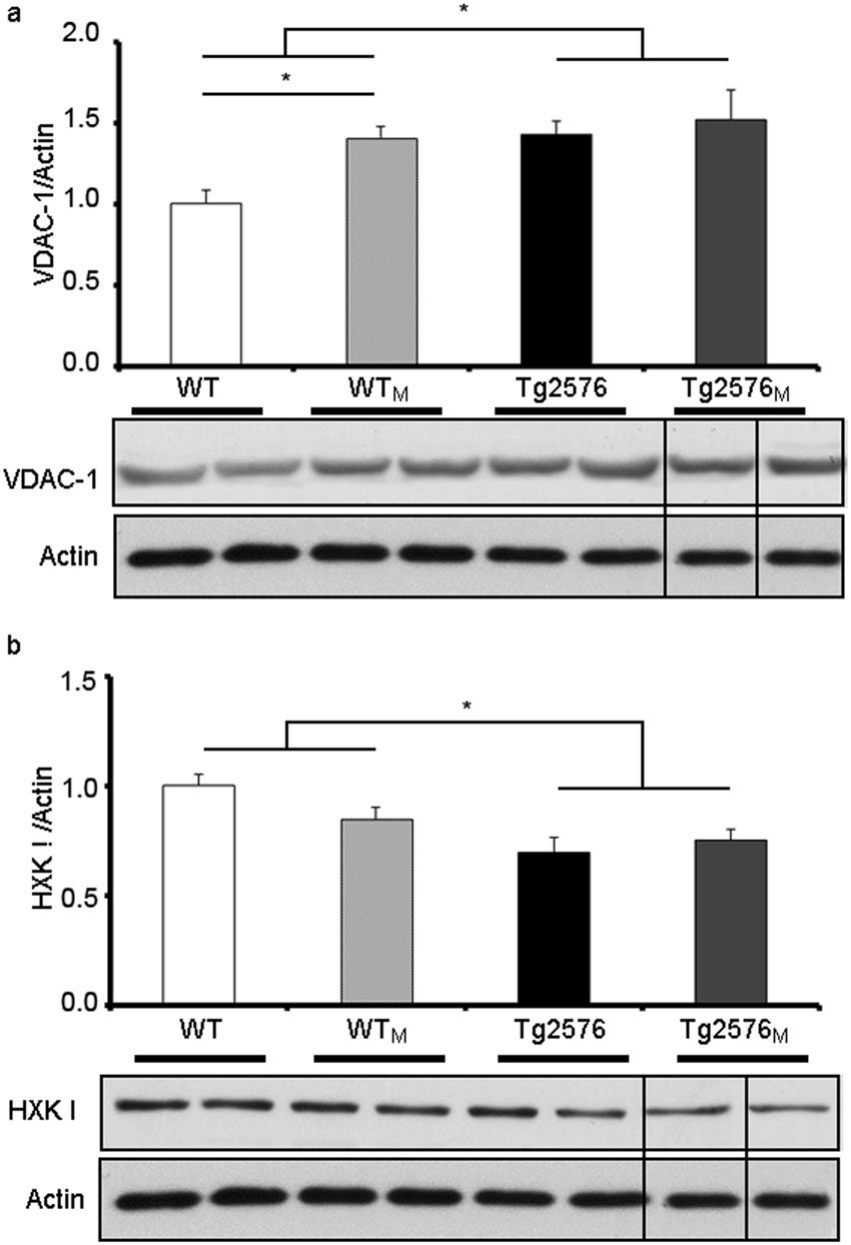
Levels of selected mitochondrial proteins in hippocampal extracts from WT, WT_M_, Tg2576 and Tg2576_M_ mice. We show a representative blot and the quantification of immunolabeled bands: VDAC-1 *versus* actin (**a**) and hexokinase type 1 (HXKI) *versus* actin (**b**). Full-length blots are presented in Supplementary Figure 6. The results (fold over control –WT-) are expressed as the mean ± SEM (n = 4). Statistical significance was analysed by two-way ANOVA with Tukey’s *post-hoc* test. *p < 0.05 respect to WT.

The two mitochondrial proteins whose levels were quantitated in hippocampal extracts were VDAC-1 (Fig. 8a), a voltage-dependent anion channel differentially expressed in the brain of AD mouse models^32^, and hexokinase type 1 (HXKI) (Fig. 8b), which is differentially expressed in AD patients^33^ and Tg2576 mice^32^. Whereas the level of HXKI was reduced in transgenic animals with no effect of maternal transmission (F_1,12_ = 8.64; p < 0.05), the level of VDAC-1 was increased in the transgenic group if compared with the non-transgenic one (F_1,12_ = 8.96; p < 0.05). It should be noted that the level of VDAC-1 in WT_M_ was also increased when compared to WT animals.

## Discussion

In order to study the effect of maternal transmission in AD we designed a particular way of generating a Tg2576-derived novel mouse model of AD. Usually the offspring of the current model is obtained from crossing healthy WT females with hAβPPswe overexpressing males. However, no studies of offspring bred from healthy WT males and hAβPPswe overexpressing females have been reported. The new model and the present findings reveal that vertical transmission from hAβPPswe-overexpressing mothers, at an age that do not display any cognitive impairment, results in accelerated cognitive deficits in Tg2576 transgenic animals. Remarkably, acquisition of characters from Tg2576 females leads to spatial memory impairment and pathological alterations in animals that do not express hAβPPswe, i.e. in the WT_M_ group.

Phosphorylation of Tau is one of the major pathological features in AD brains that also appear in the Tg2576 mouse. 8-month-old Tg2576 mice, which do not yet exhibit cognition deficits, already showed Tau pathology in terms of elevated levels of pTau and elevated levels of active Tau kinases. Furthermore, Aβ pathology in terms of amyloid accumulation was found in 8-month-old Tg2576_M_ but not in Tg2576 animals. The decrease in cytoskeletal or plasticity-related proteins is evident in young Tg2576 mice before the appearance of deficits in spatial memory. Compared to age-matched Tg2576 animals, 8-month-old Tg2576_M_ had similar or exacerbated alterations in the 15 markers analysed. In the hippocampus or cortex from both Tg2576 and Tg2576_M_ animals, Tau was heavily phosphorylated due to enhanced activity of some kinases, neural plasticity markers were down-regulated and the levels of mitochondrial VDAC-1 and HXKI were altered. Collectively, our results show that the early cognitive decline in Tg2576_M_ shares the underlying molecular determinants that are relevant for the human disease.

One remarkable finding of the present work was that mice lacking the hAβPPswe transgene but bred from young Tg2576 mothers (WT_M_) displayed biochemical traits found in aged Tg2576 and Tg2576_M_ animals. In this sense, phosphorylation of Tau was enhanced and CaMKII, Arc, Akt and AMPA GluR1 levels were reduced.

The occurrence of Aβ aggregates was confirmed using the anti-Aβ 17–24 (4G8) monoclonal antibody that reacts within the amyloid domain, as well as with the precursor forms of both human and mouse AβPP. This murine Aβ accumulation in WT_M_ mice is a striking result of this work as current murine models of amyloidosis are based on human AβPP overexpression. Nevertheless, cerebral murine Aβ accumulation has been reported in other non-transgenic mouse lines such the senescence-accelerated mouse (SAMP8)^34^ and the murine model of age-related macular degeneration^35^. Accumulation of amyloid into aggregates in the WT_M_ mouse makes it a good model to better understand the mechanisms of APP processing and Aβ aggregation.

Cognitive deficits and biochemical alterations in WT_M_ animals resemble those in Tg2576 mice, thus likely pointing to a common pathological mechanism but, of course, with differential onset. Damaged mitochondria are likely present in the ovum of hAβPPswe females. A proteomics study performed in the hippocampus of the Tg2576 showed that a large percentage of mitochondrial proteins were deregulated in 7-month-old Tg2576 mice; among them VDAC-1 and HXKI^32^. Deregulation of VDAC-1 and a clear trend towards reduced HXKI levels in WT_M_ animals suggest that ovum mitochondria may have similar alterations than hippocampal mitochondria in females overexpressing the hAβPPswe. Tg2576 animals seem to be able to cope with the extra-metabolic needs due to ER stress. Also, an adaptive variation in the level of mitochondrial proteins seems to be sufficient to keep hippocampal neurons alive^32^. Interestingly, RNA-based silencing of hAβPP, Tau, and VDAC-1 genes in human neuroblastoma cells results in improved mitochondrial function^36^. Also relevant is the possibility of mitochondrial import and degradation of Aβ^37–39^. ER signal peptide of APP may alternatively target the protein to the mitochondria and this alternative targeting depends on the structure of the nascent chain. In conditions of compromised ER import due to unstructured polypeptides hAβPP increases traffic to mitochondria^38^. Thus, it seems likely that stress caused by overexpression of hAβPPswe in ovum cells is transmitted to offspring cells and results in accelerated phenotype in Tg2576_M_ and AD-like phenotype in WT_M_ mice.

Epigenetic changes including genomic imprinting may affect LOAD risk. There are more than one hundred known imprinted genes and most of them are expressed in human brain^40^. In addition to this, maternally expressed imprinted genes in mice are expressed in cerebral cortex/hippocampal regions associated with memory tasks^41^. Thus, it is possible that silencing of imprinting of maternal alleles disrupts the cognitive function of the brain, and be a factor in neurodegenerative disorders like AD. Future studies may help to assess the relevance of epigenetics modification in AD-related maternal inheritance. In particular, epigenetic modification in mitochondrial DNA, which is only transmitted by mothers, should be taken into account. As recently reviewed^42^ this field is lacking way behind epigenetics related to nuclear DNA and more studies are, in our opinion, needed to be able to identify reliable “mitochondrial” epigenetic markers and how they may be affected in different physiological or pathological situations.

## Methods

### Generation of the mouse model

Unless otherwise stated the experiments were performed in 8-month-old mice. The genotypes of the two parental mice used were: wild type (WT) and Tg2576, which is a transgenic line that expresses in heterozygosity the human 695-aa isoform of the hAβPP containing the Swedish double mutation [(APP695)Lys670 → Asn, Met671 → Leu] driven by a hamster prion promoter^43^. WT and Tg2576 animals were generated by breeding a WT female (B6SJL genetic background) with a Tg2576 male. The homozygous Tg2576 transgene is lethal so all the Tg2576 animals used for the experiments were heterozygous. The so-called WT_M_ and Tg2576_M_ mice were generated by breeding a 8-month old Tg2576 female with a WT male (B6SJL genetic background). At 8 months of age Tg2576 females display neither amyloid plaques nor cognitive impairment in the Morris Water Maze (MWM) test. Overall, 44 animals distributed in four groups of animals were studied: WT, WT_M_, Tg2576 and Tg2576_M_.

The animals were maintained in positive pressure-ventilated racks at 25 ± 1 °C with a 12 h light/dark cycle, fed *ad libitum* with a standard rodent pellet diet (Global Diet 2014; Harlan Laboratories, Indianapolis, USA) and free access to filtered and UV-irradiated water. All animal care and experimental procedures were in accordance with European and Spanish regulations (86/609/CEE; RD1201/2005) and were approved by the Ethical Committee of the University of Navarra (no. 018/05). Behavioural studies were carried out during light time (from 9 am to 2 pm).

### Morris water maze test

8-month-old animals from WT_M_ (n = 12), Tg2576 (n = 10), Tg2576_M_ (n = 10) and WT (n = 12) groups were subjected to a hippocampus-dependent learning task in the MWM test^44^. The water maze was a circular tank (diameter 1.2 m) filled with water at 20 °C and made opaque by the addition of non-toxic white paint. Mice underwent visible-platform training for three consecutive days (6 trials/day), and were allowed to swim to a platform (10-cm diameter) located above the water level in the same position over the trials. No distal visible cues were present during this phase. Hidden-platform training was conducted over 8 consecutive days (4 trials/day). Mice had 60 s to find a hidden platform submerged 1 cm beneath the surface of the water and invisible to the mice while swimming. Several large visual cues were placed in the room to guide the mice to the hidden platform. In both visible- and hidden-platform versions of this test, mice were placed randomly in different locations each trial, facing towards the wall of the pool in order to eliminate the potentially confounding contribution of spatial cues. Mice failing to reach the platform were guided there and their position was held for 15 s until they were returned to their home cage. All trials were monitored by a camera located above the centre of the pool and connected to a SMART-LD program (Panlab S.L., Barcelona, Spain) for subsequent analysis of escape latencies, swimming speed and path length. Mice that were unable to reach the visible-platform or mice exhibiting thigmotactic patterns or persistent floating were excluded from data analyses.

### Contextual fear conditioning

The behavioral procedure involved three phases: habituation, training and testing. Four-month-old mice from WT_M_ (n = 15), Tg2576 (n = 16), Tg2576_M_ (n = 10) and WT (n = 18) groups were habituated to the conditioning box (context) for 5 min with no stimuli presented. The context consisted in a sound-proof box with white walls, light and a background noise produced by a fan. Twenty‐four hours later (training phase) mice were placed in the same context and allowed to explore for 2 min. Afterwards, a foot shock (0.3 mA) unconditioned stimulus was administered (2 s) and 30 s after mice were returned to their home cage. 24 h later mice were placed back in the conditioning chamber and allowed to explore the context for 3 min, during which freezing time was recorded (contextual long‐term memory). Freezing behavior was defined as an absence of cage displacement. Freezing scores were expressed as percentages of total freezing time. The conditioning procedure was carried out in a StartFear system (Panlab S.L., Barcelona, Spain) that allows movement recording by a high‐sensitivity Weight Transducer system and data analysis by the built-in FREEZING and STARTLE software.

### Tissue processing for immunohistochemistry

Under xylazine/ketamine anaesthesia, animals were perfused transcardially with saline and 4% paraformaldehyde in phosphate buffered saline (PBS). After perfusion, brains were removed, post-fixed in the same fixative solution for 1 h at room temperature and cryoprotected in 30% sucrose solution in PBS overnight at 4 °C. Microtome sections (30 µm-thick) were coronally cut through the entire hippocampus, collected free-floating and stored in 30% ethylene glycol, 30% glycerol, and 0.1 M PBS at −20 °C until processed.

### Immunohistochemistry

Nine free floating tissue sections comprising the hippocampal formation of three animals per group were processed for immunohistochemistry. Brain sections were washed (3 × 10 min) with a solution buffer containing PBS 0.125 M (pH 7.4), 0.5% Triton X-100 and 0.1% BSA. After washing, sections were treated with methanol and H_2_O_2_ to inhibit endogenous peroxidase activity and incubated in 70% formic acid for 7 min to expose the epitope. Sections were incubated overnight with primary antibody 4G8 (anti 18–22 Aβ amino acids; 1:500, Chemicon International, Billerica, MA, USA) or anti-pTau primary antibody AT8 (1:1000, Thermo Fisher Scientific, Rockford, USA) at 4 °C. After washing, sections were incubated sequentially with biotinylated goat anti-mouse secondary antibody (1:500, DakoCytomation, Glostrup, Denmark) for 2 h, an ABC kit immunoassay detection system (Vector Labs, Burlingame, CA, USA) for 90 min, and developed with 3,3′-diaminobenzidine (DAB) solution (Peroxidase substrate kit, Vector Labs, Burlingame, CA, USA). Sections were then washed in distilled water before dehydrating and mounting in DPX (VWR-BDH Dublin, Ireland).

Six different areas covering the entire hippocampus from two sections on anatomically comparable planes/animal were analysed. 4G8 labeling, divided into major and minor plaques, and AT8 intraneuronal immunoreactivity were blindly assessed.

### Protein extracts

Mice were killed by cervical dislocation and hippocampi were quickly dissected from the brains. Total tissue homogenates were obtained by homogenizing the hippocampus in a cold lysis buffer with protease inhibitors (0.2 M NaCl, 0.1 M HEPES, 10% glycerol, 200 mM NaF, 2 mM Na_4_P_2_O_7_, 5 mM EDTA, 1 mM EGTA, 2 mM DTT, 0.5 mM PMSF, 1 mM Na_3_VO_4_, 1 mM benzamidine, 10 µg/mL leupeptin, 400 U/mL aprotinin). After that, homogenates were centrifuged at 14.000 × g for 20 min at 4 °C and supernatants were aliquoted and stored at −80 °C. Total protein concentrations were determined using the Bio-Rad Bradford protein assay (Bio-Rad, Hercules, CA, USA).

To obtain the membrane-enriched protein fraction (P2 membrane proteins), a previously described method by Dunah *et al*. was used^45^. The hippocampi were homogenized in ice-cold Tris-EDTA buffer (10 mM Tris-HCl and 5 mM EDTA, pH 7.4), containing 320 mM sucrose and the protease and phosphatase inhibitors previously described. The tissue homogenate was centrifuged at 700 × g for 10 min. The collected supernatant was centrifuged again at 37.000 × g for 40 min at 4 °C. Finally, the pellet (P2) was resuspended in 10 mM Tris-HCl buffer (pH 7.4), containing the enzyme inhibitor mixture described above. In both cases, protein concentration was determined (Bradford assay, Bio-Rad, CA, USA) and aliquots were stored at −80 °C until use. For Western blot analysis, aliquots of the P2 membrane fraction were solubilized in denaturing conditions by adding 0.1 volumes of 20% SDS and 50% β-mercaptoethanol. The samples were incubated for 5 min at 100 °C and diluted 1:20 in 50 mM Tris-HCl (pH 9)/0.1% Triton X-100. After a centrifugation step at 37.000 × g for 10 min at 4 °C, the supernatant was stored at −80 °C.

For AβPP-derived carboxy-terminal fragments (CTFs) determination, the cortex was homogenized in a buffer containing SDS 2%, Tris-HCl (10 mM, pH 7.4), protease inhibitors (Complete Protease Inhibitor Cocktail, Roche) and phosphatase inhibitors (0.1 mM Na_3_VO_4_, 1 mM NaF). The homogenates were sonicated for 2 min and centrifuged at 100.000 × g for 1 h. Aliquots of the supernatant were frozen at −80 °C and protein concentration was determined by the Bradford method using the Bio-Rad protein assay (Bio-Rad, CA, USA).

### Immunoblotting

Protein samples from four to six mice per group were mixed with 6X Laemmli sample buffer [47% (v/v) glycerol, 0.6 M Dithiothreitol, 12% (w/v) SDS, 0.08 M Tris and bromophenol blue], boiled for 5 min and resolved onto SDS-polyacrylamide gels^46^ and transferred to nitrocellulose membrane. The membranes were blocked with 5% milk, 0.05% Tween-20 in PBS or tris-buffered saline (TBS) followed by overnight incubation with the following primary antibodies: rabbit polyclonal anti-Arc (1:1000, Millipore Corporation, Billerica, MA, USA), rabbit polyclonal anti-pGluA1 (1:1000, Millipore Corporation, Billerica, MA, USA), rabbit polyclonal anti-GluA2/3 (1:1000, Millipore Corporation, Billerica, MA, USA), mouse monoclonal anti-pGSK3β-Tyr216 (1:1000, BD Transduction Laboratories, Lexington, KT, USA), rabbit polyclonal anti-GSK3β (1:1000, Santa Cruz Biotechnology, CA, USA), mouse monoclonal anti-pTau AT8 (1:1000, Thermo Fisher Scientific, Waltham, MA USA), mouse monoclonal anti-Tau (1:5000, clone Tau 46, Sigma-Aldrich, St. Luis, MO, USA), rabbit polyclonal anti-MAP-2 (1:1000, Millipore Corporation, Billerica, MA, USA), mouse monoclonal anti-PSD95 (1:1000, Millipore Corporation, Billerica), mouse monoclonal anti-pCaMK II (1:500, Millipore Corporation, Billerica, MA, USA), mouse monoclonal anti-CaMK II (1:1000, Millipore Corporation, Billerica, MA, USA), rabbit polyclonal anti-p-Cofilin (1:1000, Cell Signaling Technology, Beverly, MA, USA), rabbit polyclonal anti-pAkt Ser473 (1:1000, Cell Signaling Technology, Beverly, MA, USA), rabbit polyclonal anti-VDAC 1 (1:1000, Millipore Corporation, Billerica, MA, USA), goat polyclonal anti-HXKI (1:200 Santa Cruz Biotechnology, CA, USA), rabbit polyclonal anti-pβ-catenin (1:1000, Cell Signaling Technology, Beverly, MA, USA), rabbit polyclonal anti-βcatenin (1:1000, Cell Signaling Technology, MA, USA), rabbit monoclonal anti-Acetyl-Histone H3 (Lys9) (C5B11) (1:2000, Cell Signaling Technology, MA, USA), rabbit polyclonal anti-Acetyl-Histone H4 (1:4000, Millipore Corporation, Billerica, MA, USA), rabbit polyclonal anti-Histone H3 (1:1500, Cell Signaling Technology,MA, USA) and mouse monoclonal anti-actin (1:20000, Sigma-Aldrich, St. Louis, MO, USA) in the corresponding buffer. Following two washes in PBS/Tween-20 or TBS/Tween-20 and one PBS or TBS alone, immunolabelled protein bands were detected by using horseradish peroxidase (HRP)-conjugated anti-rabbit or anti-mouse antibody (1:5000, Santa Cruz Biotechnology, CA. USA) following an enhanced chemiluminescence system (ECL, GE Healthcare Bioscience, Buckinghamshire, UK), and autoradiographic exposure to Hyperfilm ECL (GE Healthcare Bioscience, Buckinghamshire, UK). Quantity One software v.4.6.3 (Bio-Rad, CA, USA) was used for quantification.

For the analysis of AβPP-derived CTFs, aliquots of the protein extracts were mixed with XT sample buffer plus XT reducing agent (Bio-Rad, CA, USA) or Tricine sample buffer (Bio-Rad, CA, USA) and boiled for 5 min. Proteins were separated in a Criterion™ precast Bis-Tris 4–12% gradient precast gel (Bio-Rad, CA, USA) and transferred to nitrocellulose membranes. The membranes were blocked with 5% milk, 0.05% Tween-20 in TBS followed by overnight incubation with rabbit polyclonal anti-AβPP C-terminal (amino acids 676–695) (1:2000, Sigma-Aldrich, St Louis, MO, USA).

### BACE-1 Activity Assay

BACE-1 activity was measured using the Fluorometric Beta Secretase Activity Assay (Abcam, England, UK) according to manufacturer’s instructions. Cortical tissue (10 mg) was homogenized in 12 × (v/w) of ice-cold extraction buffer and cleared by centrifugation. 6 μl of the resulting samples were loaded into a white 384-well microplate (Optiplate, Perkin-Elmer) and the fluorogenic substrate was added in the dark. The mixture was incubated at 37 °C for one hour in the dark, and the fluorescent signal produced by cleavage of the peptide by β-secretase was measured using an Envision 2104 microplate reader (Perkin-Elmer) with 340 nm excitation and 495 nm emission wavelengths. Fluorescence was also determined in cells with vehicle, active beta secretase in extraction buffer (positive control) and beta secretase inhibitor added to the positive control (negative control).

### Data analysis and statistical procedures

The data was analysed with SPSS for Windows, version 15.0 (SPSS, Chicago, IL, USA) and unless otherwise indicated, the data is expressed as means ± standard error of the mean (SEM). Normal distribution of data was checked by the Shapiro–Wilks test. In the MWM, latencies to find the platform were examined by two-way repeated measures ANOVA test (genotype × trial) to compare the cognitive status in WT, WT_M_, Tg2576 and Tg2576_M_. Likewise, the spatial memory and the biochemical data was examined also by a two-way ANOVA test (treatment × trial) followed by *post-hoc* Tukey’s analysis. When interaction between factors was significant, single effects were analysed by one-way ANOVA followed by *post-hoc* Tukey’s test. When no significant interaction between factors was found, main effects were analysed. Student’s t-test was used in case two groups were compared.

### Data Availability

The datasets generated during and/or analysed during the current study are available from the corresponding author on reasonable request.

### Ethical approval

All animal care and experimental procedures were in accordance with European and Spanish regulations (86/609/CEE; RD1201/2005) and were approved by the Ethical Committee of the University of Navarra (number. 018/05).

## Electronic supplementary material

Supplementary Information

## References

[1] Farrer LA (1997). Effects of age, sex, and ethnicity on the association between apolipoprotein E genotype and Alzheimer disease. A meta-analysis. APOE and Alzheimer Disease Meta Analysis Consortium. JAMA.

[2] Silverman JM (1994). The Consortium to Establish a Registry for Alzheimer’s Disease (CERAD). Part VI. Family history assessment: a multicenter study of first-degree relatives of Alzheimer’s disease probands and nondemented spouse controls. Neurology.

[3] Edland S (1996). Increased risk of dementia in mothers of Alzheimer’s disease cases: Evidence for maternal inheritance. Neurology.

[4] Ehrenkrantz D (1999). Genetic epidemiological study of maternal and paternal transmission of Alzheimer’s disease. Am. J. Med. Genet..

[5] Mosconi L (2007). Maternal family history of Alzheimer’s disease predisposes to reduced brain glucose metabolism. Proc. Natl. Acad. Sci. USA.

[6] Wooten GF (1997). Maternal inheritance in Parkinson’s disease. Ann Neurol.

[7] Honea RA, Swerdlow RH, Vidoni ED, Goodwin J, Burns JM (2010). Reduced gray matter volume in normal adults with a maternal family history of Alzheimer disease. Neurology.

[8] Mosconi L (2011). Reduced mitochondria cytochrome oxidase activity in adult children of mothers with Alzheimer’s disease. J. Alzheimers. Dis..

[9] Mosconi L (2010). Maternal transmission of Alzheimer’s disease: prodromal metabolic phenotype and the search for genes. Hum Genomics.

[10] Cedazo-Minguez A, Winblad B (2009). Biomarkers for Alzheimer’s disease and other forms of dementia: clinical needs, limitations and future aspects. Exp. Gerontol..

[11] Nunomura A (2001). Oxidative damage is the earliest event in Alzheimer disease. J. Neuropathol. Exp. Neurol..

[12] Lovell MA, Soman S, Bradley MA (2011). Oxidatively modified nucleic acids in preclinical Alzheimer’s disease (PCAD) brain. Mech. Ageing Dev..

[13] Markesbery WR, Kryscio RJ, Lovell MA, Morrow JD (2005). Lipid peroxidation is an early event in the brain in amnestic mild cognitive impairment. Ann. Neurol..

[14] Luo J, Shi R (2005). Acrolein induces oxidative stress in brain mitochondria. Neurochem. Int..

[15] Kuhla B (2007). Effect of pseudophosphorylation and cross-linking by lipid peroxidation and advanced glycation end product precursors on tau aggregation and filament formation. J. Biol. Chem..

[16] Lovell MA, Xie C, Markesbery WR (2001). Acrolein is increased in Alzheimer’s disease brain and is toxic to primary hippocampal cultures. Neurobiol. Aging.

[17] Calingasan NY, Uchida K, Gibson GE (1999). Protein-bound acrolein: A novel marker of oxidative stress in Alzheimer’s disease. J. Neurochem..

[18] Axelman K, Basun H, Winblad B, Lannfelt L (1994). A Large Swedish Family with Alzheimer’s Disease with a Codon 670/671 Amyloid Precursor Protein Mutation: A Clinical and Genealogical Investigation. Arch. Neurol..

[19] Cuadrado-Tejedor M (2013). Age-related mitochondrial alterations without neuronal loss in the hippocampus of a transgenic model of Alzheimer’s disease. Curr. Alzheimer Res..

[20] Jacobsen JS (2006). Early-onset behavioral and synaptic deficits in a mouse model of Alzheimer’s disease. Proc. Natl. Acad. Sci..

[21] Dickey CA (2008). Akt and CHIP coregulate tau degradation through coordinated interactions. Proc. Natl. Acad. Sci..

[22] Ryder J, Su Y, Ni B (2004). Akt/GSK3beta serine/threonine kinases: evidence for a signalling pathway mediated by familial Alzheimer’s disease mutations. Cell Signal.

[23] Mulot SFC, Hughes K, Woodgett JR, Anderton BH, Hanger DP (1994). PHF-tau from Alzheimer’s brain comprises four species on SDS-PAGE which can be mimicked by *in vitro* phosphorylation of human brain tau by glycogen synthase kinase-3β. FEBS Lett..

[24] Aberle H, Bauer A, Stappert J, Kispert A, Kemler R (1997). Beta-Catenin Is a Target for the Ubiquitin-Proteasome Pathway. EMBO J..

[25] Orford K, Crockett C, Jensen JP, Weissman AM, Byers SW (1997). Serine phosphorylation-regulated ubiquitination and degradation of beta-catenin. J. Biol. Chem..

[26] Sze C, Bi H, Kleinschmidt-DeMasters BK, Filley CM, Martin LJ (2001). N-Methyl-D-aspartate receptor subunit proteins and their phosphorylation status are altered selectively in Alzheimer’s disease. J. Neurol. Sci..

[27] Shonesy, B. C., Jalan-Sakrikar, N., Cavener, V. S. & Colbran, R. J. In *Progress in molecular biology and translational science* **Volume 122**, 61–87 (Elsevier Inc, 2014). 10.1016/B978-0-12-420170-5.00003-9 24484698

[28] Matus A (1988). Microtubule-Associated Proteins: Their Potential Role in Determining Neuronal Morphology. Annu. Rev. Neurosci..

[29] Luo L (2002). Actin Cytoskeleton Regulation in Neuronal Morphogenesis and Structural Plasticity. Annu. Rev. Cell Dev. Biol..

[30] Ie EV (2010). The Arc of synaptic memory. Exp. Brain Res..

[31] Minamide LS, Striegl AM, Boyle JA, Meberg PJ, Bamburg JR (2000). Neurodegenerative stimuli induce persistent ADF/cofilin-actin rods that disrupt distal neurite function. Nat. Cell Biol..

[32] Mar C-T, Martínez V, Felipe C, Joaquín del R, Diana F (2011). Enhanced expression of the voltage-dependent anion channel 1 (VDAC1) in alzheimer’s disease transgenic mice: An insight into the pathogenic effects of amyloid-beta. J. Alzheimer’s Dis..

[33] Lynn BC, Wang J, Markesbery WR, Lovell MA (2010). Quantitative changes in the mitochondrial proteome from subjects with mild cognitive impairment, early stage, and late stage Alzheimer’s disease. J. Alzheimers. Dis..

[34] Yamaguchi Y, Saito K, Matsuno T, Takeda K, Hino M (2012). Effects of ZSET1446/ST101 on Cognitive Deficits and Amyloid ^|^beta; Deposition in the Senescence Accelerated Prone Mouse Brain. J. Pharmacol. Sci..

[35] Ding J-D (2008). Targeting age-related macular degeneration with Alzheimer’s disease based immunotherapies: anti-amyloid-beta antibody attenuates pathologies in an age-related macular degeneration mouse model. Vision Res..

[36] Manczak M, Reddy PH (2013). RNA silencing of genes involved in Alzheimer’s disease enhances mitochondrial function and synaptic activity. Biochim. Biophys. Acta - Mol. Basis Dis..

[37] Anandatheerthavarada HK, Biswas G, Robin M, Avadhani NG (2003). Mitochondrial targeting and a novel transmembrane arrest of Alzheimer’s amyloid precursor protein impairs mitochondrial function in neuronal cells. J. Cell Biol..

[38] Pfeiffer NV (2013). Structural features within the nascent chain regulate alternative targeting of secretory proteins to mitochondria. EMBO J..

[39] Pinho CM, Teixeira PF, Glaser E (2014). Mitochondrial import and degradation of amyloid-beta peptide. Biochim. Biophys. Acta.

[40] Barbaux S (2012). A genome-wide approach reveals novel imprinted genes expressed in the human placenta. Epigenetics.

[41] Davies W, Isles AR, Wilkinson LS (2005). Imprinted gene expression in the brain. Neurosci Biobehav Rev.

[42] Stimpfel M, Jancar N, Virant-Klun I (2018). New Challenge: Mitochondrial Epigenetics?. Stem Cell Rev. Reports.

[43] Hsiao K (1996). Correlative memory deficits, Abeta elevation, and amyloid plaques in transgenic mice. Science.

[44] Morris R (1984). Developments of a water-maze procedure for studying spatial learning in the rat. J. Neurosci. Methods.

[45] Dunah AW (2000). Alterations in subunit expression, composition, and phosphorylation of striatal N-methyl-D-aspartate glutamate receptors in a rat 6-hydroxydopamine model of Parkinson’s disease. Mol. Pharmacol..

[46] Laemmli UK (1970). Cleavage of structural proteins during the assembly of the head of bacteriophage T4. Nature.

