# Supplementary material for: Maternal imprinting on cognition markers of wild type and transgenic Alzheimer’s disease model mice
Source: Sci Rep. 2018 Apr 24;8:6434. doi: 10.1038/s41598-018-24710-7 (PMC5915602; doi:10.1038/s41598-018-24710-7)
Supplement: Supplementary file 1 — Supplementary Information [file 41598_2018_24710_MOESM1_ESM.pdf]

**Title**

Maternal imprinting on cognition markers of wild type and transgenic Alzheimer's disease model mice

**Authors**

Marta Zamarbide[a,*], Francisco Gil-Bea[a], Paul Bannenberg[a], Eva Martínez-Pinilla[a], Juan Sandoval[e], Rafael Franco[b,c,+], Alberto Pérez-Mediavilla[b,d,+]

**Author's address:**

[a] Neuroscience Department, Center for Applied Medical Research (CIMA). University of Navarra. Pamplona. Spain.

[b] Laboratory of Molecular Neurobiology. Department of Biochemistry and Molecular Biomedicine. Faculty of Biology. University of Barcelona. Barcelona. Spain.

[c] Centro de Investigación en Red, Enfermedades Neurodegenerativas (CIBERNED). Instituto de Salud Carlos III. Spain.

[d] Department of Biochemistry and Genetics. School of Sciences. University of Navarra

[e] Laboratory of personalized medicine. Epigenomics Unit. Medical Research Institute La Fe. Valencia. Spain

* zamarbidemarta@gmail.com

[+]These authors contribute equally to this work

**Supplementary figure S1**

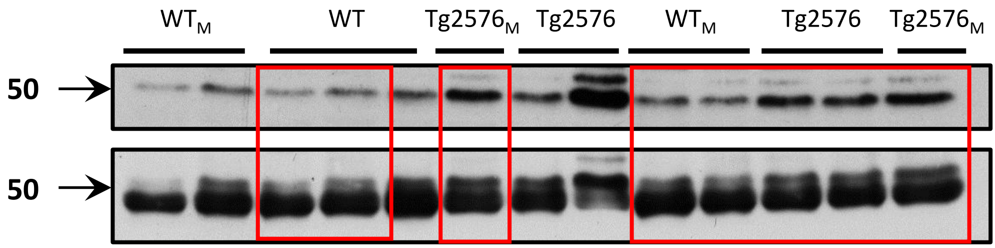


pTau and Tau expression in hippocampal membrane extracts from WT, $WT_M$, Tg2576 and $Tg2576_M$ mice

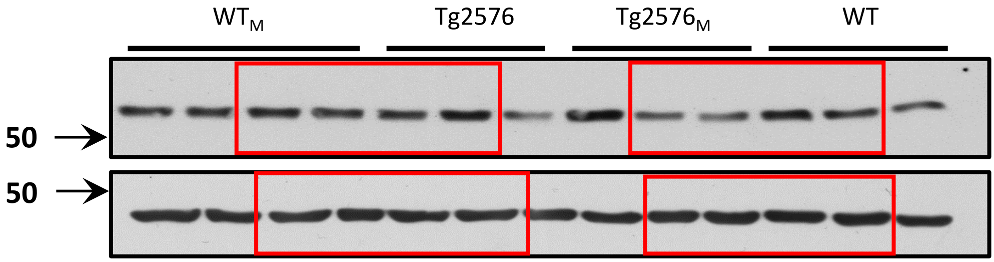


pAkt and actin expression in hippocampal membrane extracts from WT, $WT_M$, Tg2576 and $Tg2576_M$ mice

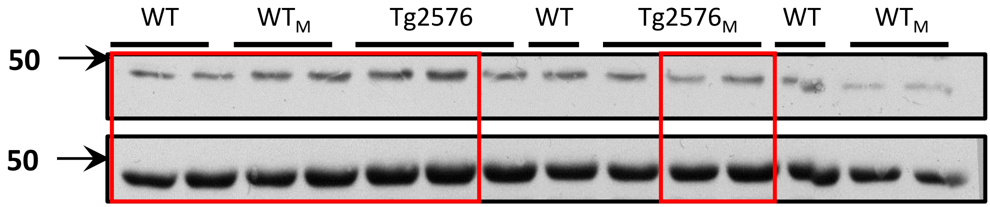


pGSK3β and GSK3β expression in hippocampal membrane extracts from WT, $WT_M$, Tg2576 and $Tg2576_M$ mice

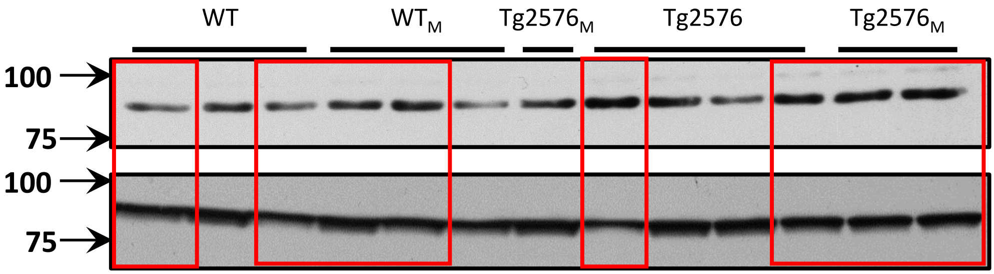


pβ-catenin and β-catenin expression in hippocampal membrane extracts from WT, $WT_M$, Tg2576 and $Tg2576_M$ mice

**Supplementary figure S2**

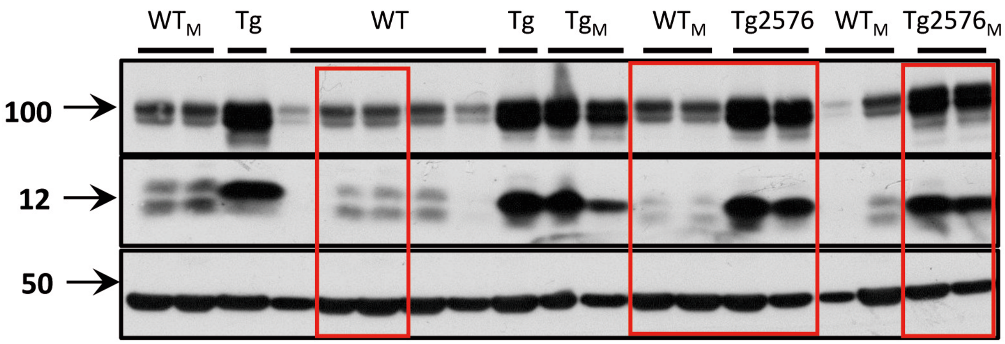


AβPP, C99, C83 and actin expression in cortical extracts from WT, WT$_M$, Tg2576 and Tg2576$_M$ mice

**Supplementary figure S3**

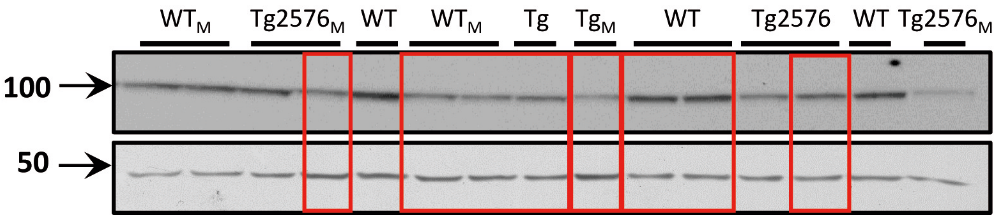


pGluA1 and actin expression in cortical extracts from WT, WT$_M$, Tg2576 and Tg2576$_M$ mice

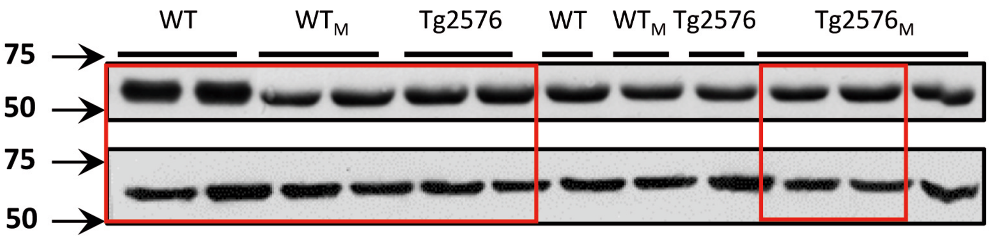


pCaMKII and CaMKII expression in cortical extracts from WT, WT$_M$, Tg2576 and Tg2576$_M$ mice

**Supplementary figure S4**

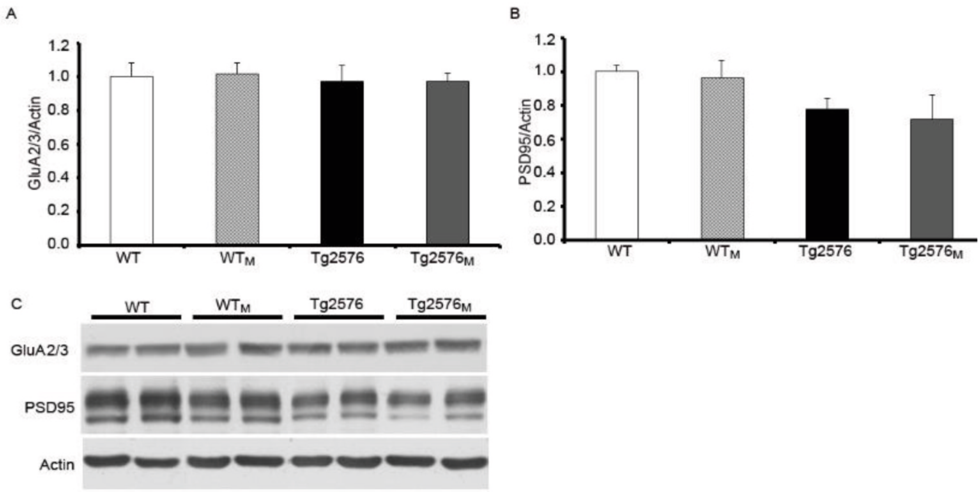


Effect of maternal transmission of non-genetic material on synaptic plasticity markers. The levels of synaptic markers in total protein cortical preparations from WT, WT$_M$, Tg2576 and Tg2576$_M$ mice were analyzed by Western blot. We show a representative blot and the quantification of immunoreactive bands. The results (fold over control –WT-) are expressed as the mean ± SEM (n=6). Statistical significance was analyzed by two-way ANOVA. (A) Quantification of AMPA subunit GluA2/3 *versus* actin. (B) Quantification of cytoskeletal protein PSD95 *versus* actin. (C) Representative blots of AMPA subunit GluA2/3, PSD5 and actin.

**Supplementary figure S5**

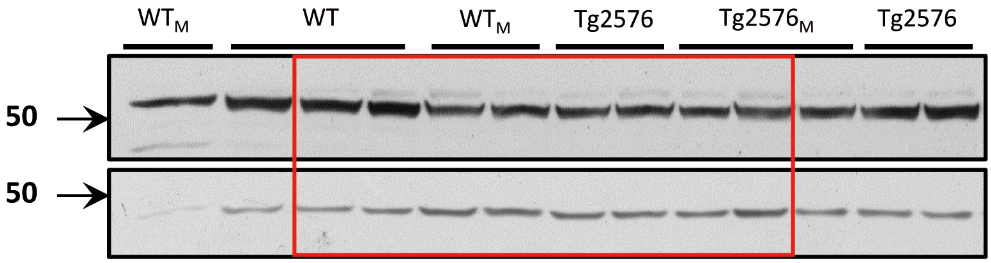


Arc and actin expression in cortical extracts from WT, WT$_M$, Tg2576 and Tg2576$_M$ mice

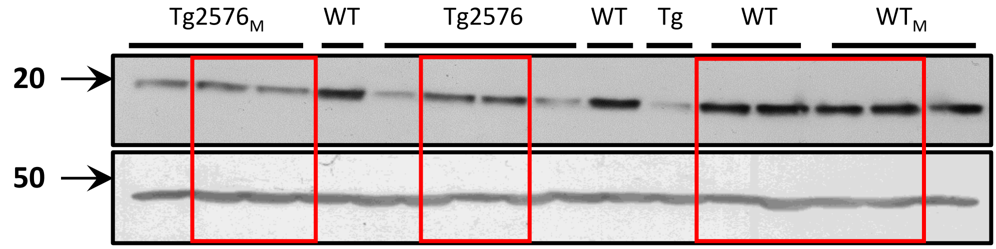


pCofilin and actin expression in cortical extracts from WT, $WT_M$, Tg2576 and $Tg2576_M$ mice

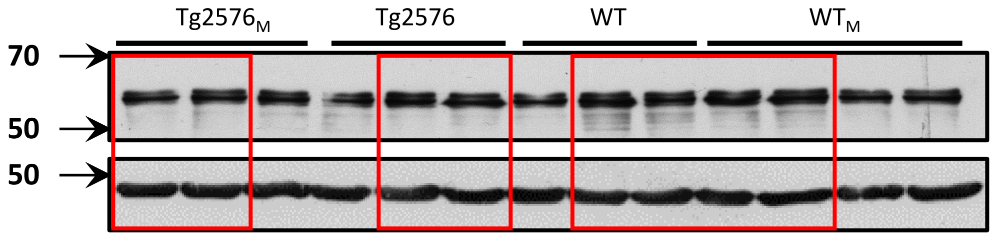


MAP2 and actin expression in cortical extracts from WT, $WT_M$, Tg2576 and $Tg2576_M$ mice

**Supplementary figure S6**

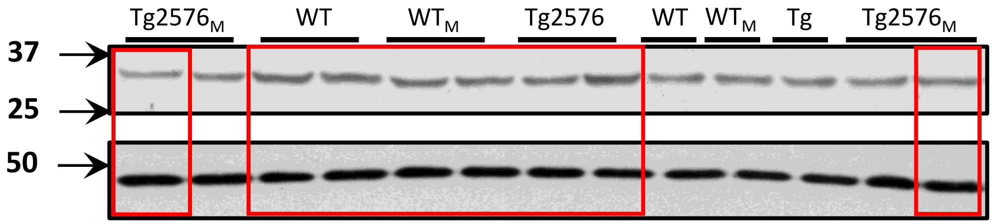


VDAC1 and actin expression in hippocampal extracts from WT, $WT_M$, Tg2576 and $Tg2576_M$ mice

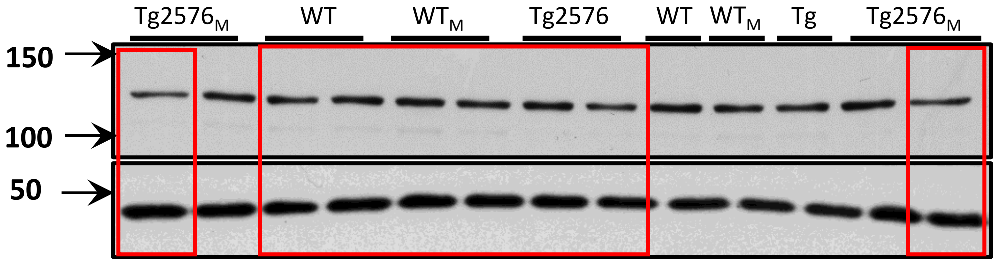


HXK I and actin expression in hippocampal extracts from WT, $WT_M$, Tg2576 and $Tg2576_M$ mice
